# Supplementary material for: Knowledge, attitudes, and practices toward Mpox among laboratory professionals in Zambia: A cross-sectional study
Source: PLoS One. 2025 Nov 3;20(11):e0335365. doi: 10.1371/journal.pone.0335365 (PMC12582439; doi:10.1371/journal.pone.0335365)
Supplement: S1 File — (PDF) [file pone.0335365.s001.pdf]

Monkey Pox

You are being invited to take part in a research study. Before you decide whether or not to participate, it is important that you understand why the research is being done and what it will involve. Please read the following information carefully.

Study title: **Assessing the knowledge, attitudes, and practices related to the Monkeypox virus among healthcare professionals in Zambia.**

This study aims to assess the knowledge of the Healthcare professionals in Zambia regarding monkeypox, and their attitude towards possible explanations of how the virus emerge. Your participation will help inform future public health interventions and educational campaigns.

Your participation in this study is entirely voluntary. Participants will not receive any allowances or incentives for taking part in this study. You may choose not to participate or to withdraw from the study at any point without giving a reason and without any negative consequences.

If you agree to participate, you will be asked to complete a questionnaire that takes approximately 10–15 minutes. The questionnaire includes questions about your knowledge, attitudes, and beliefs regarding human monkeypox and its emergence.

Principal Investigators:
David Chisompola +260968848607

* Indicates required question

1. By ticking the box below and clicking “Next,” you confirm that: *

   1. You have read and understood the study information above.

   2. You voluntarily agree to take part in this research.

   3. You are aged 18 years or older.

   Check all that apply.

   ☐ I agree to participate in this study

Personal Information

2. Age: *

Mark only one oval.

- 18–24 years
- 25–34 years
- 35–44 years
- 45–54 years
- 55–64 years
- 65 years and above

3. Gender *

Mark only one oval.

- Male
- Female
- Prefer not to say

4. Level of Education: *

Mark only one oval.

- Diploma
- Degree
- Masters
- Doctorate
- Other

5. Healthcare Professional: *

Mark only one oval.

Medical Doctor
Clinical Officer/Medical Licentiate
Nurse and Midwives
Lab Technologist/Scientist
Environmental Health Officers
Pharmacist
Public Health Specialists / Epidemiologists
Other: ____________________

6. Years of experience *

Mark only one oval.

< 1 year
1 - 5 years
6 - 10 years
> 11 years

7. Current Workplace Setting *

Mark only one oval.

Public Hospital
Private Hospital
Health Centre/Clinic
Research or Academic institution
Other: ____________________

8. **Institution name** *

____

9. Province of Practice *

Mark only one oval.

- Central Province
- Copperbelt Province
- North Western Province
- Northern Province
- Muchinga Province
- Southern Province
- Eastern Province
- Lusaka Province
- Luapula Province
- Western Province

10. Location *

Mark only one oval.

- Urban
- Rural

Section 1: Knowledge on Monkeypox (Mpox)

11. Have you heard of Monkeypox (Mpox) before? *

Mark only one oval.

- Yes
- No
- Maybe

12. What is the causative agent of Monkeypox? *

Mark only one oval.

- Virus
- Bacteria
- Parasite
- Fungi

13. What are the common modes of Monkeypox transmission? (Select all that apply) *

Check all that apply.

- Direct contact with lesions
- Airborne transmission
- Contact with infected animals
- Sexual transmission

14. What are the common symptoms of Monkeypox? (Select all that apply) *

Check all that apply.

- Fever
- Skin rash
- Swollen lymph nodes
- Diarrhoea

15. Is Monkeypox a zoonotic disease? *

Mark only one oval.

- Yes
- No

16. What is the incubation period of Monkeypox? *

Mark only one oval.

- 1 - 3 days
- 5 - 21 days
- 30 - 60 days
- Not sure

17. Is there an effective vaccine available for Monkeypox? *

Mark only one oval.

- Yes
- No
- Maybe

18. What are the potential complications of Monkeypox? (Select all that apply) *

Check all that apply.

- [ ] Pneumonia
- [ ] Encephalitis
- [ ] Severe skin infections
- [ ] None of the above

19. Can Monkeypox be diagnosed through laboratory testing?

Mark only one oval.

- Yes
- No

20. Which of the following groups are considered at high risk for severe complications from mpox? (Select all that apply.) *

Check all that apply.

- [ ] Healthy young adults
- [ ] Immunocompromised individuals (HIV)
- [ ] Children under 5 years old
- [ ] Pregnant women

21. Which of the following sample types is most appropriate for the diagnosis of Mpox?

Mark only one oval.

- ( ) Blood sample
- ( ) Saliva sample
- ( ) Skin lesion swab (e.g., vesicle or pustule fluid, scab)
- ( ) Urine sample

22. Which of the following is the most appropriate laboratory test for confirming a Mpox diagnosis?

Mark only one oval.

- ( ) Rapid antigen test
- ( ) Real-time PCR (RT-PCR)
- ( ) Enzyme-Linked Immunosorbent Assay (ELISA)
- ( ) Blood culture

Attitudes Towards Monkeypox (Mpox)

23. Do you believe Monkeypox is a serious public health threat in Zambia? *

Mark only one oval.

- Yes
- No
- Maybe

24. How concerned are you about the risk of Monkeypox infection in healthcare settings? *

Mark only one oval.

- Very concerned
- Somewhat concerned
- Not concerned

25. Do you think healthcare professionals should receive specialized training on Monkeypox management? *

Mark only one oval.

- Yes
- No
- Maybe

26. Do you believe the Hospitals are adequately prepared to handle a Monkeypox outbreak? *

Mark only one oval.

- Yes
- No
- Maybe

27. How confident are you in your ability to diagnose and manage Monkeypox cases? *

Mark only one oval.

- Very confident
- Somewhat confident
- Not confident at all

28. Do you support the mandatory vaccination of healthcare workers against Monkeypox if a vaccine is available? *

Mark only one oval.

- Yes
- No
- Maybe

29. Do you think people with Monkeypox should be isolated to prevent the spread of the disease? *

Mark only one oval.

- Yes
- No
- Maybe

30. How do you feel about the stigma associated with Monkeypox? *

Mark only one oval.

- A major concern
- A minor concern
- Not concern

31. Do you believe Monkeypox can be effectively controlled through public health interventions? *

Mark only one oval.

- Yes
- No
- Maybe

32. Should Monkeypox surveillance and reporting systems be strengthened in Zambia? *

Mark only one oval.

- Yes
- No
- Maybe

Practices Related to Monkeypox (Mpox)

33. Have you ever attended a training or workshop on Monkeypox? *

Mark only one oval.

- Yes
- No
- Maybe

34. How often do you use personal protective equipment (PPE) when handling patients with suspected infectious diseases? *

Mark only one oval.

- Always
- Sometimes
- Rarely
- Never
- N/A

35. Do you regularly update yourself on emerging infectious diseases like Monkeypox? *

Mark only one oval.

- Yes
- No
- Maybe

36. Have you ever encountered a suspected Monkeypox case in your healthcare facility? *

Mark only one oval.

- Yes
- No
- Maybe
- N/A

37. If you suspect a Monkeypox case, what would be your first step? *

Mark only one oval.

- Report to a senior doctor or infection control team
- Isolate the patient immediately
- Prescribe antibiotics
- Not sure

38. Do you follow infection prevention and control (IPC) guidelines when managing patients with infectious diseases? *

Mark only one oval.

- Yes
- No
- Maybe
- N/A

39. How frequently do you educate patients and colleagues about Monkeypox prevention? *

Mark only one oval.

- Often
- Occasionally
- Rarely
- Never

40. Have you ever participated in Monkeypox surveillance or research? *

Mark only one oval.

- Yes
- No
- Maybe

41. Do you know the reporting procedure if you identify a suspected Monkeypox case? *

Mark only one oval.

- Yes
- No
- Maybe

42. If a Monkeypox outbreak occurs in your facility, do you feel prepared to handle cases? *

Mark only one oval.

- Yes
- No
- Not sure

43. In your opinion, what strategies or interventions can be implemented to improve Mpox surveillance and prevention in Zambia? *

Thank you for taking time to answer questions.

This content is neither created nor endorsed by Google.

Google Forms
